# Development of an Adsorbing System Made of DMS-1 Mesh Modified by Amino Groups to Remove Pb(II) Ions from Water

**DOI:** 10.3390/ma13081914

**Published:** 2020-04-18

**Authors:** Viviana Palos-Barba, Cecilia Lugo-Nabor, Rodrigo R. Velázquez-Castillo, Dora Alicia Solís-Casados, Carmen L. Peza-Ledesma, Eric M. Rivera-Muñoz, Rufino Nava, Barbara Pawelec

**Affiliations:** 1 División de Investigación y Posgrado, Facultad de Ingeniería, Universidad Autónoma de Querétaro, Centro Universitario, Querétaro 76010, Qro., Mexico; vvplbr@gmail.com (V.P.-B.); rodrigo.velazquez@uaq.mx (R.R.V.-C.); 2 División de Investigación y Posgrado, Facultad de Química, Universidad Autónoma de Querétaro, Centro Universitario, Querétaro 76010, Qro., Mexico; cecilia_lugo_n@hotmail.com; 3 Centro Conjunto de Investigación en Química Sustentable, UAEM-UNAM, Km 14.5, Carretera Toluca-Atlacomulco, San Cayetano, Toluca 50200, Estado de México, Mexico; solis_casados@yahoo.com.mx; 4 Centro de Física Aplicada y Tecnología Avanzada, Universidad Nacional Autónoma de México, Departamento de Nanotecnología, A.P. 1–1010, Querétaro 76010, Qro., Mexico; carmenpez@gmail.com (C.L.P.-L.); emrivera@fata.unam.mx (E.M.R.-M.); 5 Instituto de Catálisis y Petroleoquímica, CSIC, Cantoblanco, 28049 Madrid, Spain; bgarcia@icp.csic.es

**Keywords:** disordered mesoporous silica, mesoporous adsorbent, heavy metal removal, water purification

## Abstract

Water pollution by heavy metals represents several health risks. Conventional technologies employed to eliminate lead ions from residual or drinking water are expensive, therefore an efficient and low-cost technique is required and adsorption processes are a good alternative. In this work, the goal was to determine the adsorption capacity of a Disordered Mesoporous Silica 1 material (DMS-1) functionalized with amino groups, for Pb(II) ions removal. DMS-1 was prepared by sol-gel method and the incorporation of amino groups was performed by ex-situ method. As the source of amine groups, (3-Aminopropyl) triethoxysilane (APTES) was used and three different *x*NH_2_/DMS-1 molar ratios (0.2, 0.3, 0.4) were evaluated. In order to evaluate the incorporation of the amino group into the mesopore channels, thermal and structural analysis were made through Thermogravimetric Analysis (TGA), nitrogen adsorption–desorption at 77 K by Specific Brunauer–Emmett–Teller (SBET) method, Fourier Transfer Infrared spectroscopy (FTIR), Scanning Electron Microscopy (SEM), X-Ray Diffraction (XRD) and X-Ray Photoelectron Spectroscopy (XPS). The higher Pb(II) ions removal was achieved with the 0.3 molar proportion of *x*NH_2_/DMS-1 reaching 99.44% efficiency. This result suggests that the functionalized material can be used as an efficient adsorbent for Pb(II) ions from aqueous solution.

## 1. Introduction

Lead is a heavy metal that has produced considerable interest due to the health risks it causes, even at low concentrations [1,2]. Due to anthropogenic activities like paint manufacturing processes, pottery glazes, lead soldering, batteries and the oil refining industry [3,4,5,6], lead is released to the environment becoming a threat to human health, entering the body mainly through ingestion or inhalation [4,7,8,9]. Exposure to this metal causes damage to the brain, kidneys, liver and nervous system. In addition, lead poisoning causes a physical developmental delay in infants and children [10,11,12,13]. Consequently, public health policies are necessary to prevent lead poisoning in underdeveloped and developing countries [14]. In México, the Secretary of Health established a maximum permissible concentration of 0.025 mg/L for lead in drinking water [15]. 

There are several techniques to remove lead ions from drinking water and wastewater, the most common being filtration, ion exchange and reverse osmosis [16,17]. However, these techniques do not entirely remove the metal, need vast amounts of energy and generate toxic sludge [9]. Compared to other methods, adsorption by a mesoporous material is simple, economical and effective [18]. In addition, due to the reversibility of the adsorption process, the adsorbing systems can be regenerated [19]. Therefore, there is a necessity to continue the research on adsorbing materials with high adsorption capacities and efficiency [20,21,22]. According to the IUPAC classification, materials that have pores with diameters ranging from 2 to 50 nm are called mesoporous [23]. These materials have unique [24] textural properties: large specific surface and large pore volume, and they are thermally stable, which makes them versatile for many applications [25,26,27,28,29,30]. Within this group, the Disordered Mesoporous Silica 1 (DMS-1) is a material developed by our group that possesses a high surface area (600–1000 m^2^/g) and has a spherical pore shape with a wormhole morphology [31,32]. In particular, the highly porous DMS-1 material can be used to support molecules, as reported before for catalysts [33], like NH_2_ groups to promote an accurate mass transfer in the pore network of NH_2_/DMS-1. 

The influence of the sorbent morphology on its sorption capacity is well known. For example, it was found that the initial adsorption rate onto platelet mesoporous adsorbents was rapid and faster than the rates of rod-like and fiber-like morphologies [34]. However, contrary to ordered mesoporous silica materials such as SBA-15, MCM-1 etc., the use of Disordered Mesoporous Silica 1 (DMS-1) as sorbents is scarcely investigated [35]. This prompted us to investigate the adsorption capacity and structural stability of ammonia-modified DMS-1 material for Pb(II) removal from an aqueous solution in order to compare the adsorption capacity of those materials with amine-functionalized SBA-15 and SBA-16 sorbents studied by us previously [32,36]. Our preliminary study demonstrated that the DMS-1 functionalized with thiol groups exhibit the suitable textural properties for its use as an adsorbent to remove Hg(II) ions from water [35].

The adsorption of Pb(II) cations by mesoporous silica functionalized with amino groups has been investigated previously [32,36,37,38,39,40]. It was clearly demonstrated the higher affinity of those materials for negatively charged Pb(II) ions with respect to bare mesoporous silica [32,36,37,38,39,40]. This behavior can be explained taking into account that different forms of the metal ions exists in the pH ranges [40]. In such case, the functionalization of silica surface with amino groups changes the point of zero charge (PZC) of adsorbent allowing the optimization of the van der Waals electrostatic interaction between Pb(II) ions and the amino groups grafted onto silica surface [32,36]. In the other words, there is the pH depending linkages of the protonated amino groups with Pb(II) ions leading to formation of the -Si-NH_2⋯_Pb(II) structures [32,36].

Within this scenario, the aim of this study was to develop an effective adsorbing system made of DMS-1 functionalized with amino groups to remove Pb(II) ions from aqueous solutions. To attain this, the following conditions were optimized to perform the adsorption: concentration of amino group, contact time, initial pH, and temperature, regarding the effectiveness for the Pb(II) removal. Taking into account that the *x*NH_2_/DMS-1 exhibits an open pore structure and accessible adsorption sites, there are necessary conditions for a good contact between the lead ions and the adsorption sites. In addition, to evaluate the Pb^2+^ adsorption in the functionalized DMS-1, structural characterization by Fourier Transform Infrared Spectroscopy (FTIR) spectra, N_2_ adsorption-desorption isotherms, X-Ray Diffraction (XRD), Thermogravimetric Analysis-Derivative Thermogravimetry (TGA-DTG), and X-Ray Photoelectron Spectroscopy (XPS) were performed. It has been demonstrated that the main advantage of the DMS-1 sorbent was the stability of their -Si–O–Si– bonds formed between the amino and the hydroxyl groups on silica surface. Although it is commonly believed that the more amino groups bonded to the silica surface the better cations removal from an aqueous solution, the results presented in this work shows that ammonia content should be optimized to obtain the maxima of lead removal. It was concluded that the amino-functionalized DMS-1 adsorbents should be interesting and promising sorbents for heavy metal ions removal from aqueous solutions.

## 2. Materials and Methods 

### 2.1. Synthesis of the DMS-1 Mesoporous Silica

Disordered Mesoporous Silica 1 (DMS-1) was synthesized according to the sol-gel method proposed by Zhao (1998) and Fuertes (2004) [41,42]. The structure-directing agent pluronic triblock copolymer F127 (EO_106_–PO_70_–EO_106_, 99%, Aldrich) was used as the surfactant and tetraethyl orthosilicate (TEOS, 98%, Aldrich) as the silica source. In a typical synthesis procedure, the triblock copolymer was dissolved in a mixture of deionized water and 2M hydrochloric acid (HCl, 36.5–38.0% JT Baker) solution, and the mix was stirred for 1 hour, after that time, the required quantity of TEOS was added to the mixture at 30 °C and it was kept under magnetic stirring for 24 h. The mixture was subsequently transferred to polypropylene bottles and they were heated at 80 °C for 48 h. Then, the obtained solid was filtered, washed with deionized water, dried at 110 °C for 18 hours and finally, it was calcined in an air atmosphere at 550 °C for 4 h to remove the organic molecules [32].

### 2.2. Synthesis of Amine-Functionalized DMS-1

The functionalization of the internal surface of mesopores in DMS-1 has been achieved by the ex-situ method. The amino groups were attached to the internal mesoporosity in DMS-1 using a solution of 3-Aminopropyl-triethoxysilane (APTES NH_2_(CH_2_)_3_Si(OEt)_3_, 99%, Aldrich) in ethanol (absolute, Aldrich) [43]. The materials were prepared varying the concentration of APTES to obtain samples with the following APTES: TEOS molar compositions: 0.2:1.0, 0.3:1.0, and 0.4:1.0. The NH_2_-modified samples were labeled hereafter as 0.2 NH_2_/DMS-1, 0.3 NH_2_/DMS-1, and 0.4 NH_2_/DMS-1. One gram of DMS-1 was dispersed into a solution made of APTES-ethanol for 30 min at 30 °C. After that time, deionized water was gradually added and lastly, the liquid suspension was put to stirr for additional 30 min at 30 °C. Water was incorporated in order to perform hydrolysis of the alkoxide groups of APTES. The added water amount was twice the amount required to completely hydrolyze the APTES content. Finally, the solids obtained were dried at room temperature and posteriorly at 50 °C for 24 h.

### 2.3. Adsorption Experiments

The efficiency of the amine-functionalized DMS-1 materials to remove Pb(II) ions from water was determined by a batch technique. To evaluate this removal efficiency, 20 mL of 100 mg/L Pb(II) aqueous solutions were put in contact with 0.1 g of the adsorbent material. The Pb(II) solution was prepared with a Pb standard (Tracert, 1000 ppm, Sigma-Aldrich).

The effects of contact time, pH [44], the temperature during the adsorption process, and the initial Pb ion concentration, on the adsorption capacity were analyzed. The batch adsorption experiments were performed at 30 °C under natural pH conditions, and stirring was performed for 1 h (100–130 rpm). The solution pH was adjusted using either a 0.1 mol/L HCl solution or a NaOH solution (JT Baker) with the same concentration. After adsorption experiments, the suspensions were filtered, the adsorbing materials were recovered and the final solution was collected. 

Lead (II) ions concentration was determined by inductively coupled plasma atomic emission spectroscopy (ICP-AES), in water samples taken before and after adsorption experiments. The effects of the parameters listed above on the adsorption capacity for functionalized DMS-1 and bare DMS-1 were evaluated. Determination of Pb(II) ions concentration was performed in an Optima 8300 spectrometer (Perkin Elmer, Waltham, MA, USA). The emission lines were used according to the standard Environmental Protection Agency (EPA) method to analyze this metal [45]. Adsorbed Pb(II) amount was calculated considering the initial and final concentrations in the aqueous solution.

### 2.4. Physico-Chemical Characterization of the Supporting Materials

The mesoporous materials were studied to know their textural properties by the nitrogen adsorption isotherms registered at 77 K using a volumetric Autosorb-IQ2 adsorption analyzer (Anton Paar QuantaTec, Boynton Beach, FL, USA) [46]. Before the adsorption experiments, each sample was dried at 150 °C for 24 h under vacuum (10^−4^ mbar) to ensure a clean, dry surface free of any loosely adsorbed species. The specific areas (SBET) of the samples were calculated via the standard Brunauer–Emmett–Teller (BET) procedure [47] using the nitrogen adsorption data collected at a relative equilibrium pressure interval of 0.03 < P/P_0_ < 0.3. The total pore volume (V_p_) was estimated from the amount of nitrogen adsorbed at a 0.99 relative pressure [48,49,50]. The pore size distributions were calculated from the adsorption and desorption branches of the corresponding nitrogen isotherm via the Barrett–Joyner–Halenda (BJH) model [51]. The thermal stability of the adsorbents was studied by thermal gravimetric analysis-derivative thermogravimetry (TGA-DTG) [52,53] using a TGA Q5000 calorimeter (TA Instruments, New Castle, USA). All TGA and DTG curves were obtained with a high-resolution on a temperature range from 25 through 800 °C with a nitrogen flow. 

The functional groups on the amine-functionalized adsorbents were studied by an Infrared Spectrum Two spectrophotometer (Perkin Elmer, Waltham, MA, USA) [54,55,56,57] and using the KBr wafer technique in the spectral region from 400 to 800 cm^−1^. Pore arrangement on DMS-1 was analyzed by low-angle X-Ray Diffraction (XRD). XRD diffractograms were obtained with a Rigaku Ultima IV diffractometer (Rigaku, Akishima, Tokyo, JAPAN) using Cu Kα (λ = 1. 5406 Å) radiation [58] in the 2θ angular range from 0.1 to 10°. Scanning Electron Microscopy (SEM) was utilized to observe the pore structure and morphology on the DMS-1 particles using a Hitachi SU8200 microscope (Hitachi High-Technologies, Tokyo, JAPAN). Secondary electrons images were recorded and an accelerating voltage of 200 kV was used in the microscope [59,60]. X-Ray Photoelectron Spectroscopy (XPS) was employed to identify the nature of chemical species on the surface of DMS-1 using a JPS 9200 XPS instrument (Jeol, Akishima, Tokyo, JAPAN) equipped with a Mg Kα radiation source (1253.6 eV). The spectrometer was operated at pass energy of 10 eV with an X-ray power of 300 W, the base pressure in the analyzing chamber was maintained in the order of 10^−8^ mbar. The binding energy was determined using the carbon C(1s) line as a reference with a binding energy of 284 eV [61]. The standard deviation in the binding energy (BE) values was +0.l eV. The high-resolution narrow-scan spectra obtained were smoothed and fitted using the Gaussian functions of the origin 92E software [62].

## 3. Results

### 3.1. Characterization of Mesoporous Silica Materials

**Figure 1:**
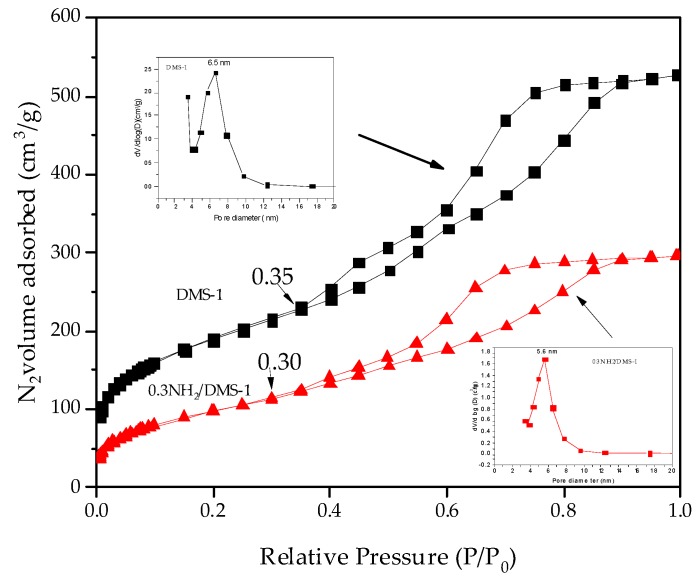
Nitrogen adsorption-desorption isotherm data of Disordered Mesoporous Silica 1 (DMS-1) adsorbent before functionalization and after functionalization with amines. Left insert: pore size distribution for DMS-1 material. Right insert: pore size distribution for amino-functionalized 0.3NH_2_/DMS-1 material.

Textural properties were obtained from the nitrogen adsorption-desorption isotherms. Specific surface area, total pore volume, and average pore size were determined for all samples. Nitrogen adsorption-desorption isotherms for the bare DMS-1 and 0.3NH_2_/DMS-1 are shown in Figure 1. Both samples exhibited a type IV adsorption-desorption isotherm, which is characteristic of mesoporous materials with 6–8 nm pore diameter [42,63,64,65]. The hysteresis loop present in the range from 0.40 to 0.90 P/P_0_ is a H1 type, distinctive from materials with pore diameters between 6 and 8 nm, and is typical for wormhole structured DMS-type materials [66]. The isotherm of pure DMS-1 showed a desorption branch with two stages indicating the presence of a structure with open pores. In contrast, the adsorbent 0.3NH_2_/DMS-1 showed a decrease in the desorption first stage, suggesting that the NH_2_ groups are attached to the internal surface of the DMS-1 pores. 

Texture properties from the materials are summarized in Table 1. DMS-1 and 0.3NH_2_/DMS-1 samples showed a uniform and narrow pore size distribution, which had average size values of about 6.5 and 5.6 nm, respectively. This result corroborates that the modification reaction of DMS-1 using a molar concentration of 0.3 of APTES did not produce significant damage to the structure of the material. Therefore, the surface area and the total pore volume of the modified material reduced their values significantly compared to those determined for the pure DMS-1. The decrease in the pore volume value in the 0.3NH_2_/DMS-1 is a consequence of the functionalization process. The nitrogen adsorption–desorption study also confirms that the analyzed samples are DMS-type materials.

**Figure 2:**
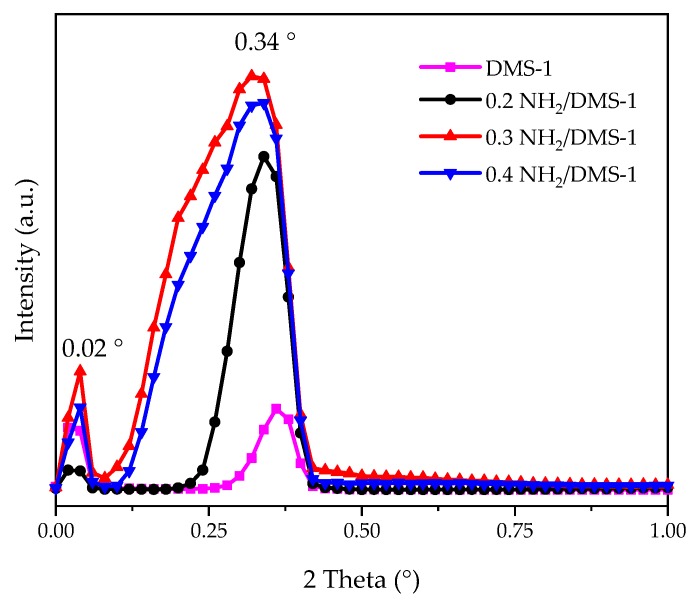
Low-angle XRD patterns of bare DMS-1 and *x*NH_2_/DMS-1 adsorbents in the range from 0 to 1° in 2θ. All samples presented reflections at 0.02 and 0.34°.

Figure 2 shows the small-angle X-ray diffractogram of DMS-1, 0.2NH_2_/DMS-1, 0.3NH_2_/DMS-1 and 0.4NH_2_/DMS-1 materials. For the DMS-1 sample, two Bragg reflections were observed at the values of 0.02° and 0.34°, which indicate there are two different pore sizes repeated in the mesh structure, corresponding to pores formed among particles (0.02°), and the textural pores; both in the mesopore range. The functionalized materials 0.2NH_2_/DMS-1, 0.3NH_2_/DMS-1 and 0.4NH_2_/DMS-1 showed similar Bragg reflections at 0.02° and about 0.34°. However, the reflections about 0.34° suffered a slight displacement toward smaller angle values, their intensity and width increased as a function of the amino group concentration. For 0.4NH_2_/DMS-1 adsorbent, this reflection was shorter and thinner than that corresponding to 0.3NH_2_/DMS-1; these differences could be produced by saturation of functionalizing molecules in the open side of pores, complicating the diffusion of these molecules within the pore.

**Figure 3:**
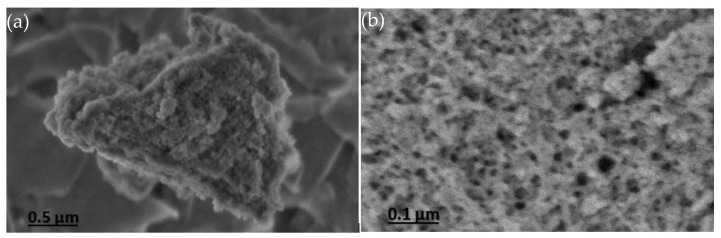
SEM images of the 0.3NH_2_/DMS-1 adsorbent showing (**a**) irregular shape of the particle, and (**b**) the disordered structure of the mesoporos.

SEM images of the 0.3NH_2_/DMS-1 material are shown in Figure 3. The images showed irregular particles with no defined morphology, as can be seen in Figure 3a. Irregular surface and the disordered arrangement of pores are visible in Figure 3b.

### 3.2. FTIR Analysis

**Figure 4:**
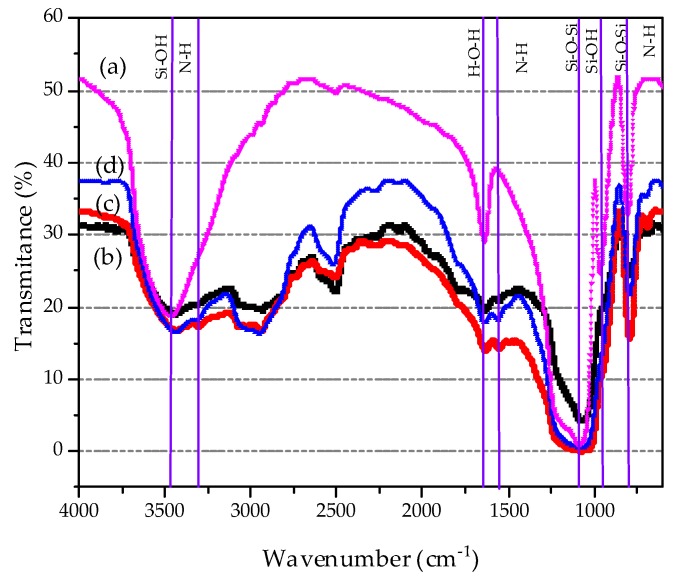
Fourier-Transformed Infrared spectra of (**a**) bare DMS-1 material, (**b**) 0.2NH_2_/DMS-1, (**c**) 0.3NH_2_/DMS-1, and (**d**) 0.4NH_2_/DMS-1

The FTIR spectra of pure DMS-1 and the adsorbent materials with different amino concentration are displayed in Figure 4, where a typical silica spectrum is observed. For all samples, the bands at 1087 and 808 cm^−1^ are clearly visible, which are related to the asymmetric and symmetric stretching vibrations of Si–O–Si bonds, respectively [67,68,69,70]. The 950 cm^−1^ band could be associated with stretches of Si-O- or Si-OH groups, probably hydrogen-bonded to adsorbed H_2_O molecules. The band at 1631 cm^−1^ is correlated to the O–H deformation bending vibration of physically absorbed water. The broad band centered at 3400 cm^−1^ is due to silanol groups having cross hydrogen bonding which interacts with the adsorbed water molecules [71,72]. It is also observed the characteristic bands for vibrations of the C–H bonds (2926, 1927 and 1882 cm^−1^) of methylene groups [73,74]. In the amino-functionalized samples, the wide band about 3290 cm^−1^ in the range of 2700–3400 cm^−1^ is attributed to NH_2_ stretching vibration. Noticeably, the 0.3NH_2_/DMS-1 exhibits higher intensity of this band than 0.4NH_2_/DMS-1 counterpart suggesting the largest surface exposure of NH_2_- groups. The weak N–H bending vibration at 950 cm^−1^ and the symmetric –NH_2_ bending vibration around 1561 cm^−1^, confirms the incorporation of amino groups [18].

### 3.3. Thermogravimetric Analysis (TGA-DTG)

**Figure 5:**
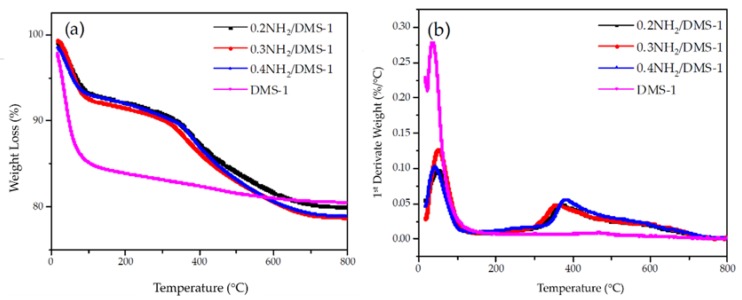
Weight loss analysis: (**a**) Thermogravimetric Analysis, and (**b**) Derivative Thermogravimetry for the bare DMS-1 and the amino-functionalized adsorbents

Thermogravimetric analysis of bare DMS-1, 0.2NH_2_/DMS-1, 0.3NH_2_/DMS-1 and 0.4NH_2_/DMS-1 mesoporous materials are shown in Figure 5a,b in a gradual weight loss up to 800 °C. Compared with 19.4% weight loss of the DMS-1 sorbent measured during heating, the 0.2NH_2_/DMS-1, 0.3NH_2_/DMS-1 and 0.4NH_2_/DMS-1 samples exhibit weight losses of 19.93%, 21.04% and 21.04% respectively. The profiles (Figure 5a,b being TGA and DTG, respectively) of bare DMS-1 silica sample, 0.2NH_2_/DMS-1, 0.3NH_2_/DMS-1 and 0.4NH_2_/DMS-1 showed the presence of inflection points at 45 and 250 °C that corresponds to dehydration and dehydroxylation of the materials, respectively [75,76]. Finally, the amino-functionalized mesoporous samples 0.2NH_2_/DMS-1, 0.3NH_2_/DMS-1 and 0.4NH_2_/DMS-1 showed the inflection points at nearly 369, 359 and 317 °C as can be seen in Figure 5b, which are coincident with the decomposition temperature of the amino functional group. Above 217 °C, thermal stability can be seen for amino groups, as the boiling point of APTES, [76] suggesting that the DMS-1 material can be stable at the temperature of decomposition of the species in the surface. Thus, TG characterization demonstrated that the main advantage of the DMS-1 sorbent is the stability of their -Si–O–Si– bonds formed between the amino and the hydroxyl groups on silica surface.

### 3.4. Determination of Amino Concentration in DMS-1 Adsorbent

**Figure 6:**
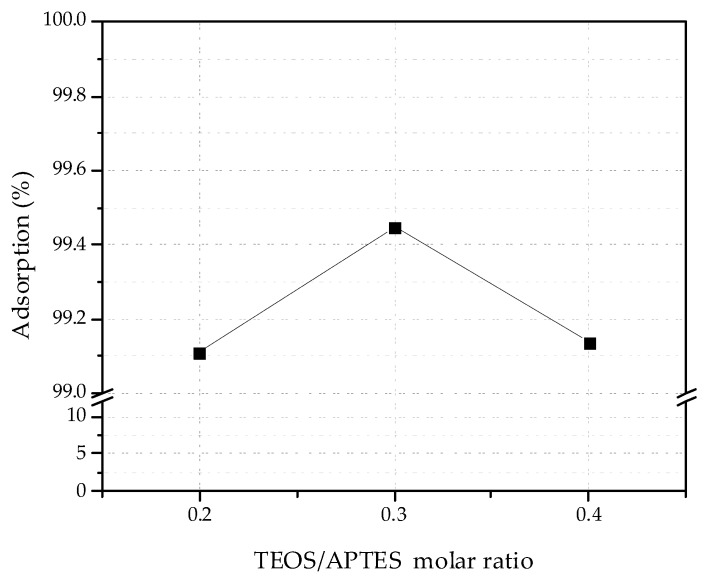
Effect of NH_2_ groups concentration for the adsorption of Pb(II) ions onto *x*NH_2_/DMS-1 materials. Adsorption conditions: initial volume 20 mL; 0.10 g of adsorbents; contact time 60 min; temperature solution 30 °C

In order to establish the appropriate adsorption conditions for Pb(II) removal with amine-functionalized materials, several tests were made at natural pH and 30 °C for 1 h. The adsorption of Pb(II) ions for each adsorbent 0.2NH_2_/DMS-1, 0.3NH_2_/DMS-1 and 0.4NH_2_/DMS-1 is shown in Figure 6. The adsorption accomplished a maximum at the 3.3 molar ratio TEOS/APTES (0.3NH_2_/DMS-1) with 99.44% removal. Thus, although it is commonly believed that the more amino group bonded to the silica surface the better cations removal from an aqueous solution, the results presented in this work shows that amount of amino groups should be optimized to obtain the maxima of lead removal.

### 3.5. Adsorption Tests

**Figure 7:**
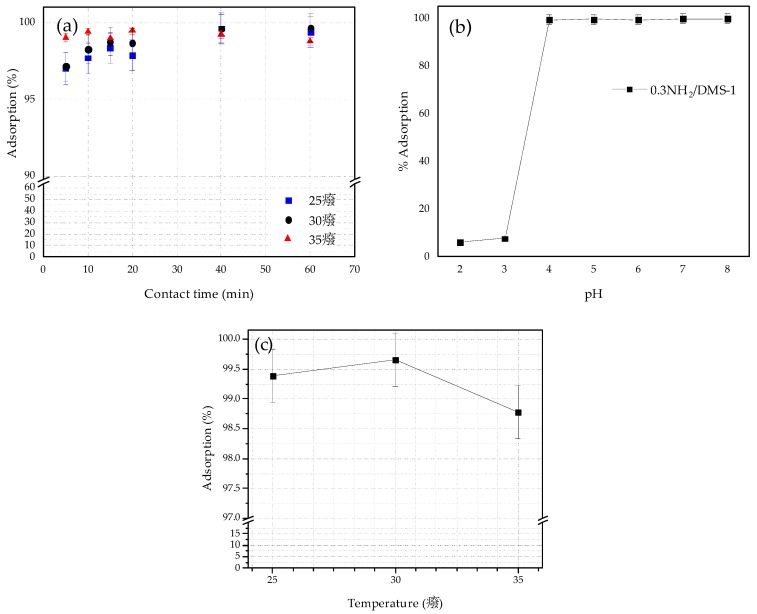
The effect on the Pb(II) ions adsorption onto 0.3NH_2_/DMS-1 of: (**a**) Contact time from 5 to 60 min at pH 5 and 25, 30 and 35 °C, (**b**) pH ranging from 2 to 8, at 30 °C for 60 min, and (**c**) Temperature at pH 5 for 60 min. Conditions: 20 mL of 100 ppm of Pb(II) ions solution, 0.10 g of adsorbent

The adsorption of Pb(II) ions by the selected adsorbent (0.3NH_2_/DMS-1) was studied by a batch operation, the experiments were performed to determine the effects of contact time, the pH value, adsorption temperature and initial lead ion concentration in solution. The effect of contact time on the removal efficiency of Pb(II) within the range from 0 to 60 min under pH 5 at 25, 30 and 35 °C is observed in Figure 7a. The removal efficiency increased gradually when increasing temperature and the adsorption rate reached equilibrium in less than 60 min. Thus, the optimal contact time was considered 60 min for the subsequent experiments.

The influence of the pH on the adsorption capacity was studied and the results are exposed in Figure 7b, ranging from 2 to 8 pH values at 30 °C. The initial Pb(II) concentration was 100 mg/L, and the adsorption was tested for 60 min. The solution pH value is an important parameter affecting adsorption properties, its effects can be associated with metal speciation in solution [77], changes of the ionic forms of the functional groups on the silica surface, and the competition effects of hydrogen ions with metal ions [78,79] and other mechanisms. The predominant species of lead ions in solution (pH < 6.0) are mainly Pb(II) and PbOH^+^, and with the increasing of pH, less Pb(II) but more PbOH^+^ species are present in the solution [80]. The species of Pb(OH)_2_ occur and precipitate in solution with pH > 6 [78,80], which will conduct to wrong experimental data. The lowest removal efficiency was carried out at pH 3 with 7.72%, otherwise, at pH values of 4, 5 and 6, the adsorption achieved 99.35%, 99.66% and 99.12%, respectively. Thus, the adsorption capacity of 0.3NH_2_/DMS-1 for Pb (II) adsorption was improved with an increase of the pH of solution from 2 to 5. Taking in mind that the surface of bare silica is acidic due to presence of silanol groups (its isoelectric point (IEP) is close to pH 2) whereas the surface of the silica grafted with aminopropyl groups is more basic [81], the deposition of the positively charged Pb^2+^ cations on the negatively charged surface of adsorbate increases above the PZC point of this adsorbate. Thus, the possible explanation of an increase of Pb(II) removal with an increase of pH of solution are two: (i) the amount of protonated amino groups decreased (–NH_2_ groups) due to their de-protonation forming negatively charged sites, and/or (ii) the higher electrostatic attraction at pH of 5. At higher pH, the surface of 0.3 NH_2_/DMS-1 has a negative charge because of the high concentrations of –OH ions in the aqueous solution, favorable for the adsorption of Pb(II). Considering the study by Huang et al. [82], the Pb^2+^ and Pb(OH)^+^ species are dominant species at pH < 6 and pH > 7.5, respectively. The hydrolysis of Pb^2+^ to form Pb(OH)^+^ and Pb(OH)_2_ occurs at pH 3.7 and 6.8, respectively. Therefore, the best removal of lead by 0.3NH_2_/DMS-1 was performed at pH = 5.

The optimization of the DMS-1 surface archived with the 0.3 molar proportion of _x_NH_2_/DMS-1 can be explained considering the attachment of ligands as the point-wise linkage to the silica surface, while at higher molar proportion of amino groups (*x*NH_2_/DMS-1) the amino groups are attached as the loop structures bonded with the silica, as shown as shown in Figure 2. If the ligands form the loop it blocks two silanols. Molecular models reveal that the loop structure is sufficiently long to shield the additional, third silanol, as shown in Figure 3. This allows one molecule of octamethylcyclotetrasiloxane (OMCTS) to block three silanols and that also explains quite well, a low-molecular coverage obtained for OMCTS modified surface.

Figure 7c presents the effects of temperature on the removal efficiency of Pb(II) by 0.3NH_2_/DMS-1 under pH 5 for 60 min, the removal efficiency increased gradually with the rise of temperature from 25 to 30 °C, but decreased from 99.57% to 98.63% with the intensification in the temperature from 30 to 35 °C. This decrease suggests that the adsorption process is exothermic. The study reveals that the adsorption process of heavy metal ions was improved at 30 °C possibly by the exothermic electrostatic interactions [83].

**Figure 8:**
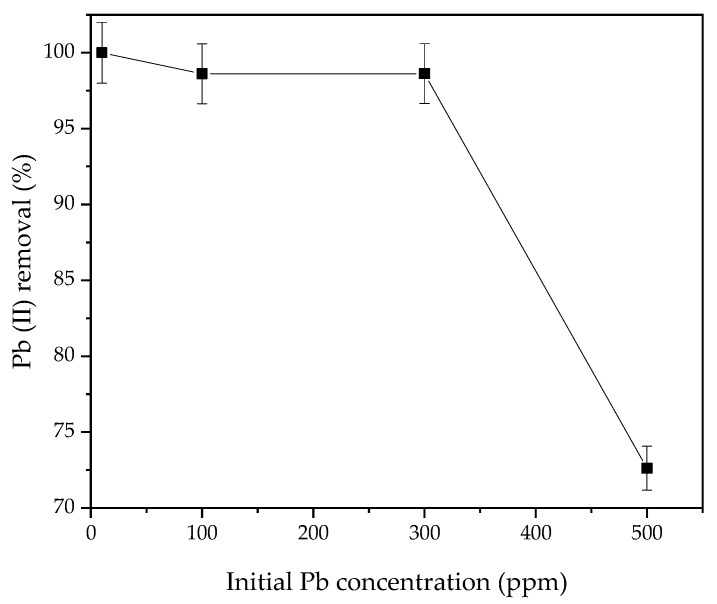
Effect of initial Pb(II) concentration on the adsorption on the 0.3NH_2_/DMS-1 material. Conditions: 20 mL of 100 ppm of Pb(II) ions solution, 0.10 g of adsorbent; contact time 60 min, temperature solution 30 °C; solution pH 5

In order to explain the mechanism responsible for Pb(II) removal from aqueous solutions by adsorption on functionalized siliceous DMS-1 materials, the effect of the initial concentration of Pb(II) ion adsorption on the most optimized 0.3 NH_2_/DMS-1 adsorbent was investigated. The effect of initial metal concentration on the adsorption rate was analyzed within the range 10–500 mg/L at pH 5 and 30 °C for 60 min, as shown in Figure 8. It can be seen from the figure that the removal percentage is nearly 100% throughout the initial metal ions concentration range 10–100 mg/L and the removal decreases with the increase in initial Pb(II) concentration. At lower initial Pb(II) ion concentrations, sufficient adsorption sites are available for adsorption of the Pb(II) ions. However, at higher concentrations, the amount of Pb(II) ions is relatively higher compared to the availability of adsorption sites. Hence, the removal of Pb(II) depends on the initial metal ions concentration. At higher initial lead ion concentration (>300 ppm), more Pb(II) was left in solution due to the saturation of binding site. Desorption of the adsorbed Pb(II) ions from 0.3NH_2_/DMS-1 could be effectively realized using acid treatment, as it was demonstrated for ZnCl_2_-MCM-41 sorbent regenerated with HNO_3_ [84]. Similarly, the recycling experiments showed that the SBA-15 sorbent could be regenerated by acid treatment without altering its properties [82].

### 3.6. X-ray Photoelectron Spectroscopy (XPS)

**Figure 9:**
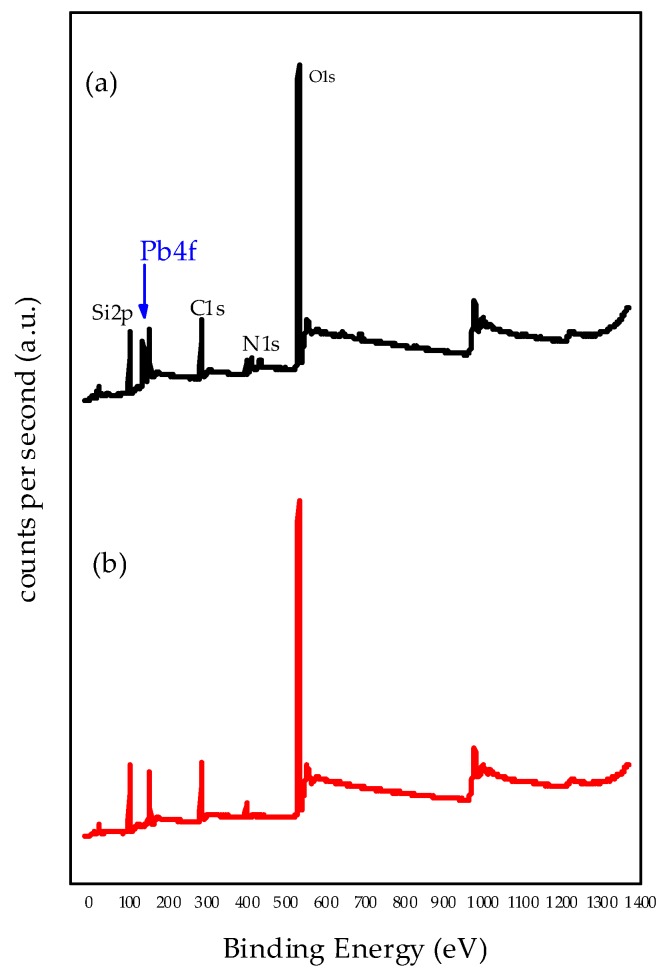
X-ray Photoelectron Spectroscopy wide-scan spectrum of 0.3NH_2_/DMS-1 adsorbent from solution with 100 ppm of Pb(II) ions: (**a**) After lead ion adsorption, and (**b**) before lead ion adsorption

The most effective adsorbent was studied by XPS techniques to elucidate the nature of adsorption sites. The introduction of the Pb(II) ions onto 0.3NH_2_/DMS-1 adsorbent was examined by the XPS wide-scan spectrum in Figure 9 with the 0.3NH_2_/DMS-1 sample before and after lead ion adsorption from the solution containing 100 ppm of Pb(II) ions. The binding energies of Si2p, C1s, O1s, N1s, and Pb4f_7/2_ core electrons and surface atomic ratios (N/Si and Pb/N) are compiled in Table 2. All adsorbents showed a peak at around 530.6 eV, which is assigned to the typical oxygen bonds in Si–O–Si [85]. The C1s peak at 283.17 eV is due to a C-Si bond usually for the presence of carbon contaminating the surface [86], and binding energies at 102.88 eV and at 102.92 eV are attributed to Si-O bonds [87,88,89]. The N1s peaks observed at 399.3 and 401 eV are related to the -NH_2_ and protonated -NH_3_^+^ bonds respectively. These species were detected before Pb(II) adsorption and also after adsorption, although the –NH_2_ gets protonated, there are still enough concentration of -NH_2_ able to coordinate with the Pb^2+^ ions over the pair of free electrons. The atoms of nitrogen located in the surface of the silicon resulted in an atomic ratio of 0.13, and the Pb/N revealed an atomic ratio of 1.38.

**Figure 10:**
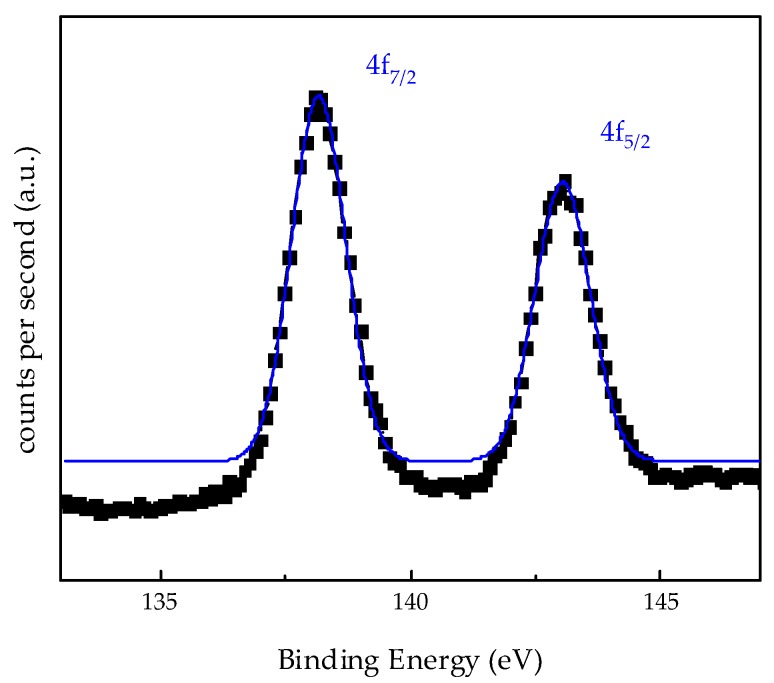
X-ray Photoelectron Spectroscopy spectra of Pb4f core levels after lead ion adsorption on 0.3NH_2_/DMS-1 adsorbent from solution with Pb(II) initial concentration 100 ppm

Figure 10 shows XPS Pb4f spectra after Pb(II) adsorption from solutions containing 100 ppm. Adsorbent 0.3NH_2_/DMS-1 showed two peaks (Pb4f_7/2_ and Pb4f_5/2_) resultant from a spin-orbit doublet. Identifying the Pb4f_7/2_ core level, the binding energy at 138.17 eV was observed and it is higher than those reported for the orthorhombic PbO compound (137.2 eV) and more similar to the binding energy reported for Pb(NO_3_)_2_ (138.6 eV) [90,91,92]. Therefore, no evidence of precipitation of Pb as hydroxides was found with the photoelectron binding energies of adsorbed Pb4f_7/2_ during adsorption. 

## 4. Conclusions

NH_2_/DMS-1 systems with groups amino to remove Pb(II) ions from aqueous solutions were studied. The affinity between the synthesized materials and the heavy metal is attributed to their atomic properties and to the pH value from the solution. The optimum pH values for the removal of the lead ions by 0.3NH_2_/DMS–1 material were pH 4 to 6. Lead adsorption on amino-modified DMS-1 materials may be explained by the Van der Walls electrostatic interactions between the Pb(II) ions and the -NH_2_ surface.

## Figures and Tables

**Table 1:** Textural properties ^a^ of calcined DMS-1 before and after functionalization with NH_2_ groups.

Sample	S_BET_ (m^2^/g)	V_pmeso_ (cm^3^/g)	D_p_ (nm)
DMS-1	668	0.67	6.5
0.3 NH_2_/DMS-1	354	0.39	5.6

^a^ Specific Brunauer–Emmett–Teller surface area (SBET), total volume of mesopores (V_pmeso_) and average pore diameter (D_p_) as determined by N_2_ physisorption at 77 K.

**Table 2:** Binding energy (eV) values of core-electrons from the detected elements, their assignments and surface atomic ratios of 0.3NH_2_/DMS-1 adsorbent before and after Pb(II)

XPS Data	Before Adsorption (eV)	After Adsorption (eV)
Si 2p	102.88	102.92
O 1s	530.64	530.60
C 1s	283.17	283.17
N 1s	399.27 400.96	399.30 401.07
Pb4f_7/2_	-	138.17
	-	143.04
N/Si	0.15	0.13
Pb/N	-	1.38
